# Peripheral *vs*. Central Cannulation in Cardiac Reoperations: Technical Considerations and Outcomes

**DOI:** 10.21470/1678-9741-2019-0445

**Published:** 2020

**Authors:** Emin Can Ata, Korhan Erkanli, Mustafa Özer Ulukan, Yahya Yıldız, Halil Türkoglu, Sedat Paslı

**Affiliations:** 1 Department of Cardiovascular Surgery, Medipol Mega University Hospital, Istanbul, Turkey.; 2 Department of Anesthesiology and Reanimation, Medipol Mega University Hospital, Istanbul, Turkey.

**Keywords:** Cardiopulmonary Bypass, Survival Rate, Retrospective Studies, Reoperation, Sternotomy, Heparin, Jugular Veins, Femoral Vein, Aorta, Heart Atria, Catheterization, Erythrocytes

## Abstract

**Objective:**

To compare peripheral and central cannulation techniques in cardiac reoperation.

**Methods:**

This retrospective study included 258 patients undergoing cardiac reoperation between January 2013 and July 2018. Patients were divided into two groups according to the cannulation type. The first group included 145 (56.2%) patients operated with standard central cannulation through aorta and right atrium or bicaval cannulation. In this group, cardiopulmonary bypass was instituted after sternotomy. The second group consisted of 113 (43.8%) patients operated with peripheral cannulation through femoral artery, vein, and internal jugular vein. In this group, cardiopulmonary bypass was started before sternotomy and after systemic heparinisation. The two groups’ operative complications and postoperative outcomes were compared.

**Results:**

Procedure-related injury was higher in the central cannulation group than in the peripheral cannulation group (8.3% *vs.* 1.8%, respectively, *P*=0.038). Cardiopulmonary bypass time was shorter in the central cannulation group (*P*=0.008) and total operation time was similar between the groups (*P*=0.115). Postoperative red blood cell requirement was higher with central cannulation (*P*=0.004). Operative mortality (2.8% *vs.* 0, *P*=0.186), hospital mortality (4.3% *vs.* 2.7%, *P*=0.523), and one-year survival rate (90.3% *vs.* 94.7%, *P*=0.202) were similar between the groups.

**Conclusion:**

Peripheral cannulation reduces cardiac injury and blood transfusion in cardiac reoperation. The cannulation type does not affect postoperative complication, mortality, and one-year survival.

**Table:** 

Abbreviations, acronyms & symbols	
AVR	= Aortic valve replacement
CABG	= Coronary artery bypass grafting
CC	= Central cannulation
CPB	= Cardiopulmonary bypass
CT	= Computed tomography
EuroSCORE	= European System for Cardiac Operative Risk Evaluation
IABP	= Intra-aortic baloon pump
ICU	= Intensive care unit
IVC	= Inferior vena cava
MVR	= Mitral valve replacement
PC	= Peripheral cannulation
RBC	= Red blood cell
TEE	= Transesophageal echocardiography

## INTRODUCTION

Cardiac reoperation is a challenging process that requires a special strategy and precaution against complications at every stage of surgery. Injury to the right ventricle, decreasing of the great artery pressure, and the patent bypass graft can cause catastrophic outcomes during resternotomy and pericardial dissection. While there is no single method to eliminate complications and mortality, efforts and argues are ongoing for years^[1,2]^.

Although some studies underline the advantages of peripheral cannulation (PC)^[3,4]^, there is also a study showing that the routine use of PC is unnecessary, and standard central cannulation (CC) has good results^[3]^. In this study, we aimed to discuss technical considerations, complications, and postoperative outcomes of these two different managements of redo cardiac surgery.

## METHODS

This retrospective study was carried out after approval of the Ethics Committee of Istanbul Medipol University. The study results were accepted by the hospital authorities.

### Inclusion

A total of 258 patients undergoing cardiac reoperation with CC or PC technique between January 2013 and July 2018 at our center were included in this study.

### Exclusion

Patients with severe peripheral vascular disease operated with CC during May 2016 and July 2018 were excluded from this study due to our study design.

### Study Design

In this study, we included 258 patients undergoing redo cardiac surgery between January 2013 and July 2018 in our center. Their mean age was 70±6.3 (range 29-82) years, female and male patients were 107 (41.5%) and 151 (58.5%), respectively. During this period, two different cannulation techniques were applied for cardiopulmonary bypass (CPB). From January 2013 to April 2016, all the patients were operated with CC, there were 145 (56.2%) patients in this first group. The second group included 113 (43.8%) PC patients, from May 2016 to July 2018. In the CC group, the cannulation was performed via aorta, right atrium, or bicaval cannulation, as standard physiological fashion. In the PC group, cannulation was established through the femoral vein and artery, and internal jugular vein; CPB was initiated before sternotomy, after systemic heparinisation. The two groups’ operative variables, major complications, operative mortality, and one-year survival rate were retrospectively analyzed.

### Operative Technique

**Fig. 1:**
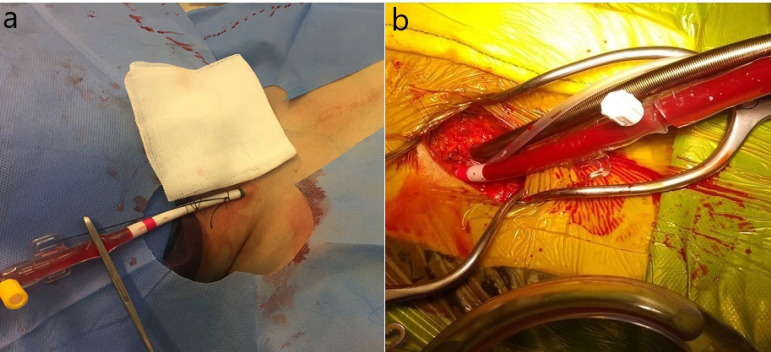
Cannulation for peripheral cardiopulmonary bypass. A) Internal jugular vein cannulation; B) femoral artery and vein cannulation.

All the operations were performed by the same surgical team with CPB under mild to moderate hypothermia (26-32 °C). After applying aortic cross-clamp, diastolic arrest was achieved by cold (4 °C) blood cardioplegia in antegrade, retrograde, or combined fashion. Resternotomy was performed by oscillating saw. In the CC group, underlying structure and pericardial dissection was performed for aortic, right atrial, or bicaval cannulation. After systemic heparinisation, CPB was started, further or full pericardial dissection was carried out if necessary. In the PC group, thorough assessments of the descending aorta and iliofemoral and internal jugular veins were conducted by doppler ultrasound and thoracic computed tomography (CT) scan before the operation. Patients with aneurysm or dissection, bilateral severe iliofemoral calcification, or stenosis were not included in this group. This special patient group was operated with CC to prevent limb ischemia, but they were excluded from the CC group if operated after April 2016 due to the predefined time period of this study. Inferior vena cava (IVC) filter existence was also accepted as exclusion criteria because of unsafe venous cannulation. After systemic heparinisation, the jugular vein was placed with Medtronic DLP femoral artery cannula (17F-21F, Minneapolis, Minnesota, United States of America) under ultrasound guide using Seldinger technique (Figure 1A). Then, a 2-3 inch of groin incision was made and the femoral artery and vein were exposed. A 5/0 polypropylen suture was placed circularly on the vein, and double parallel ‘U’ sutures on the artery^[5]^. The femoral vein was cannulated first by a suitable size Medtronic Edwards Lifesciences (18F-28F, Irvine, California, United States of America) femoral cannula, then Medtronic DLP femoral artery cannula (17F-21F, Minneapolis, MN, United States of America) was inserted between the double parallel ‘U’ suture (Figure 1B). The tips of the cannulas were identified by transesophageal echocardiography (TEE) for proper positioning, then CPB was instituted before sternotomy.

**Fig. 2:**
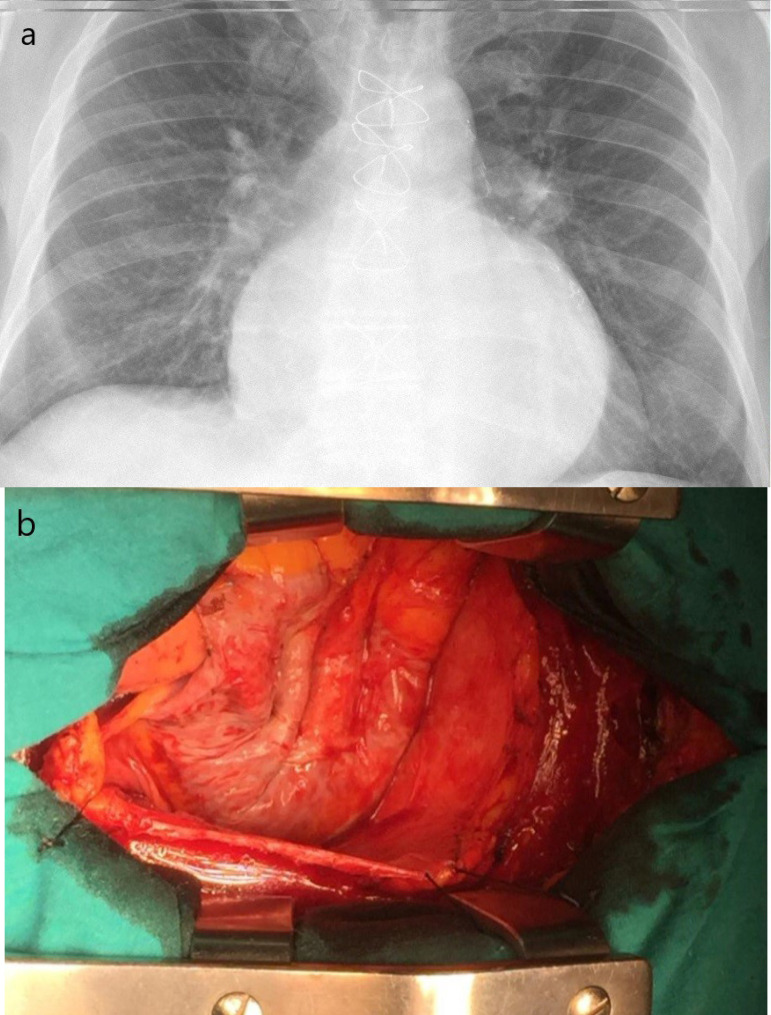
Decompression of huge right atrium with peripheral cardiopulmonary bypass. A) Preoperative chest X-ray; B) decompressed right atrium.

Pericardial dissection was started from the diaphragmatic surface of the heart, then continued to the right atrium and aorta with electrocautery and Metzenbaum scissors. In mitral and aortic procedures, the left ventricular side was not dissected, being left untouched (Figure 2A). If patent bypass graft occurred, we started the pericardial dissection from the aortic proximal anastomosis site, then continued distally toward/over the graft (Figure 2B). De-airing was conducted via aortic root needle under TEE in both groups.

### Data Collection

The patients’ medical records were collected in a predefined standard form and transferred to the computer. Baseline characteristics, operative variables, major complications such as reexploration, postoperative renal failure and neurological and cognitive dysfunction, postoperative wound complication, perioperative mortality, and one-year survival were obtained from the Turkish online programs Pusula or Nucleus.

### Statistical Analysis

Statistical analysis was performed with IBM SPSS Statistics software (SPSS Inc. Chicago Illinois, United States of America), version 24.0. The normal distribution of the variables was examined by histogram graphs and the Kolmogorov-Smirnov test. Mean and standard deviation values were used to present descriptive analyses. Pearson’s chi-squared and Fisher’s exact tests were compared with 2´2 tables. When normally distributed (parametric) variables were evaluated among the groups, Student’s ***t*** -test was used. Mann-Whitney U test was used to evaluate nonparametric variables. ***P*** -values < 0.05 were considered statistically significant results.

## RESULTS

There were no differences between the comorbid factors and calculated European System for Cardiac Operative Risk Evaluation - EuroSCORE II values of the two groups (Table 1). In both groups, the most frequent performed reoperation was isolated mitral valve replacement (34.5% ***vs.*** 33.6%, ***P*** =0.886), and the least performed surgery was combined valvular operation (3.4% ***vs.*** 9.7%, ***P*** =0.046). The reoperation types were not homogeneous between the two groups (Table 2).

**Table 1:** Preoperative clinical characteristics.

	CC (n=145)	PC (n=113)	*P*-value
Age (years)	68.2±5.5	66.6±7.6	0.217
Female	65 (44.8%)	42 (37.2%)	0.216
Diabetes mellitus	24 (16.6%)	17 (15.1%)	0.743
Hypertension	25 (17.2%)	22 (19.5%)	0.646
Peripheral vascular disease	12 (8.3%)	3 (2.7%)	0.069
Chronic obstructive pulmonary disease	8 (5.5%)	6 (5.3%)	0.942
Left ventricular ejection fraction (%)	50.0±8.4	48.9±8.3	0.313
EuroSCORE II	7.19±2.94	7.35±3.13	0.242
Similarity between the first and the reoperation types			
Same	82 (56.5%)	49 (43.3%)	0.036
Different	32 (22.1%)	36 (31.9%)	0.078
Both	31 (21.4%)	28 (24.8%)	0.519

CC=central cannulation; EuroSCORE=European System for Cardiac Operative Risk Evaluation; PC=peripheral cannulation

**Table 2:** Distribution of reoperation types.

	CC n (%)	PC n (%)	Total n (%)	*P*-value
Isolated MVR	50 (34.5)	38 (33.6)	88 (34.1)	0.886
Isolated CABG	38 (26.2)	17 (15.0)	55 (21.3)	0.032
Isolated AVR	26 (18.0)	12 (10.6)	38 (14.7)	0.104
Aortic procedures	19 (13.1)	23 (20.4)	42 (16.3)	0.120
CABG+valvular	7 (4.8)	12 (10.6)	19 (7.4)	0.085
Combined valvular	5 (3.4)	11 (9.7)	16 (6.2)	0.046
Total	145 (56.2)	113 (43.8)	258 (100)	

AVR=aortic valve replacement; CABG=coronary artery bypass grafting; CC=central cannulation; MVR=mitral valve replacement; PC=peripheral cannulation

CPB time was shorter in the CC group than in the PC group (120±26.7 ***vs.*** 125±31, respectively, ***P*** =0.008). Total operation time (198±43 ***vs.*** 202±47, ***P*** =0.115) and aortic cross-clamp time (87.4±20.4 ***vs.*** 91.3±20.6, ***P*** =0.139) were similar between the groups. During resternotomy and pericardial dissection, there was a higher injury rate in the CC group than in the PC group (8.3% ***vs.*** 1.8%, respectively), and this was statistically significant (odds ratio 5.2, 95% confidence ınterval 1.1-22.9, ***P*** =0.038). The PC technique reduced procedure-related injury. Postoperative bleeding was similar between the groups (***P*** =0.204), but red blood cell (RBC) transfusion rates were lower in the PC group (***P*** =0.004). Pre-strenotomy CPB reduced blood wastage. In this study, no difference was found between the two methods in terms of major complication rates (Table 3). Prolonged ventilation and intra-aortic baloon pump requirement were also similar between the groups. Postoperative inotrope requirement was very high in both groups, but statistically insignificant (63.0% ***vs.*** 56.6%, *P*=0.267) (Table 4).

**Table 3:** Operative variables, blood transfusions, and major complications.

	CC (n=145)	PC (n=113)	*P*-value
Cardiopulmonary bypass time (min)	120±26.7	125±31	0.008
Aortic cross-clamp time (min)	87.4±20.4	91.3±20.6	0.139
Operation time (min)	198±43	202±47	0.115
Procedure-related injury	12 (8.3%)	2 (1.8%)	0.038*
Right ventricle	3	1	
Right atrium	2	1	
Coronary vasculature	3	0	
Aorta	1	0	
Left innominate vein	2	0	
Inferior vena cava	1	0	
Postoperative bleeding (ml)	744±315	720±296	0.204
Red blood cell (pack)	2.9±1.89	2.6±1.85	0.004
Fresh frozen plasma (pack)	0.94±1.44	0.96±1.35	0.723
Cryoprecipitate (pack)	0.44±1.42	0.41±1.32	0.672
Platelet concentration (pack)	0.45±1.35	0.46±1.38	0.973
No blood transfusion patient	21 (14.5%)	12 (10.6%)	0.358
Reexploration	11 (7.6%)	3 (2.7%)	0.097
Renal failure**	7 (4.8%)	6 (5.3%)	0.860
Pneumonia	6 (4.1%)	3 (2.7%)	0.523
Stroke	7 (4.8%)	4 (3.5%)	0.613
Cognitive disfunction	11 (7.6%)	5 (4.5%)	0.302
Wound complications	6 (4.1%)	5 (4.4%)	0.909
Sternal	6 (4.1%)	3 (2.7%)	
Femoral	0	2 (1.8%)	

* Odds ratio 5.01; 95% confidence interval 1.1-22.9

** Defined as peak creatinine value ≥ 1.5 ´ preoperative value

CC=central cannulation; PC=peripheral cannulation

**Table 4:** Postoperative follow-up and mortality.

	CC (n=145)	PC (n=113)	*P*-value
ICU stay (hours)	33±12	29±15	0.160
Prolonged ventilation (> 24 h)	13 (9.0%)	8 (7.1%)	0.583
IABP	8 (5.5%)	4 (3.5%)	0.458
Inotrope requirement (> 6 h)	92 (63.0%)	64 (56.6%)	0.267
Length of hospital stay (days)	6.9±2.0	6.7±2.2	0.143
Operative mortality	4 (2.8%)	0	0.186
Hospital mortality (within first month)*	6 (4.3%)	3 (2.7%)	0.523
One year survival (%)	90.3	94.7	0.202

* Includes operative mortality

CC=central cannulation; IABP=intra-aortic baloon pump; ICU=intensive care unit; PC=peripheral cannulation

In the CC group, four (2.8%) operative mortalities occurred. Among them, one patient died of aortic injury right after sternotomy and another patient died of IVC injury during dissection, these two mortalities happened in the operating room; the other two patients died in the intensive care unit (ICU) because of failed repair of right ventricular injury. No operative mortality occurred in the PC group, but the difference was not statistically significant (***P*** =0.186). Hospital mortality (within one month) was also similar between the groups (4.9% ***vs.*** 2.7%, *P*=0.523). Pneumonia and low cardiac output-associated multi-organ failure were the leading cause of early postoperative mortality. One-year survival rates were excellent and similar between both groups (90.3% ***vs.*** 94.7%, ***P*** =0.202) (Table 4).

## DİSCUSSİON

The most common challenge in cardiac reoperation is undoubtedly the uneventful sternotomy. Injury of underlying cardiac structure may cause serious bleeding and hemodynamic instability during resternotomy^[6]^. Up to April 2016, all the reoperations were performed with CC in our center; since then, we completely switched to PC with the hope of further reducing operative mortality and morbidity. Kuralay E et al.^[3]^ had performed experiences with Carpentier bicaval venous cannula and significantly reduced cardiac injury and catastrophic hemorrhage. By similar way, Luciani et al.^[4]^ conducted successful peripheral CPB in selected patients before sternotomy and also reduced reentry injury. Total operation time and CPB time with PC was found to be longer in both studies. In our study, we used multiple-stage venous cannula for peripheral CPB, and unlike Luciani et al.^[4]^, we did not assign any predefined conditions for peripheral CPB. Our study showed that peripheral CPB time was longer than central CPB time, and this is similar to the abovementioned studies, but total operation time did not show a significant difference between the groups. Presternotomy peripheral CPB decreases intracardiac and great artery pressure, these facilitated easy and rapid dissection of adhesions.

Studies are suggesting that the preprocedural planning with multidetector CT is useful for determining the proximity between the sternum and underlying structure^[7,8]^. We agree with this, but as we know, it is not a tool to give adequate information about the adhesion’s severity. Yoshioka I et al.^[9]^ showed that tagged cine magnetic resonance imaging with a finite element model can predict the severity of retrosternal adhesions, however, this technique is not widely used in practice. Our routine is to perform CT angiography before aortic operations, such as Bentall or David procedures. In other cases, we consider that coronary angiography imaging performed before surgery is sufficient for the localization of patent bypass grafts, the CT angiography is unnecessary. We always started the dissection from the proximal anastomosis of the patent graft toward distally, along with the graft (Figure 2B). Left internal mammary artery graft is easy to identify with dissection starting from the apex. In this way, no graft injury occurred in our study. In selected patients, especially those with mitral or aortic pathology with pulmonary hypertension, we prefer to remove the sternal wires after sternotomy. This technique might be helpful to reduce right atrial and ventricular injury. In mitral and aortic procedure, there is no need to dissect left ventricular and apex region, this may also reduce cardiac injury and unnecessary hemodynamic compromise.

**Fig. 3:**
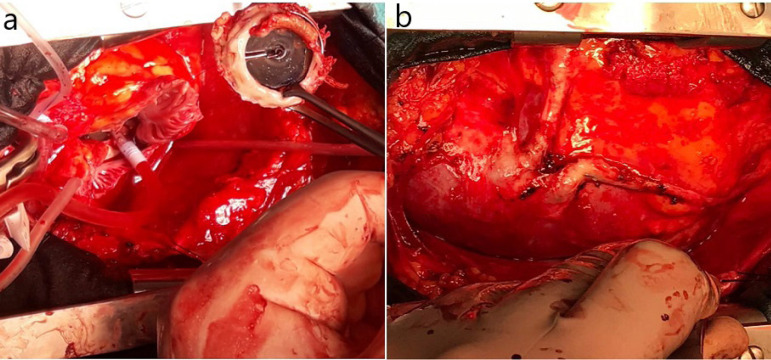
Reoperation view with peripheral cardiopulmonary bypass. A) Mitral replacement for disfunctional mitral prosthesis; B) vein graft exposure for coronary artery bypass.

In our study, less injury occurred in the PC group during sternotomy and pericardial dissection (*P*=0.038). The most important reason for this is that the decompression of the heart and the decreased pressure in major arteries allow safer sternotomy and pericardial dissection under presternotomy CPB (Figure 3). In our practice, eight out of the 12 injuries in the CC group could be successfully repaired. Aorta and İVC injured in two patients could not be repaired due to massive bleeding, and they died in the operating room. The right ventricular injury in the other two patients was repaired initially, but the bleeding reappeared at the end of the operation and pericardial patching was applied. However, prolonged CPB and massive blood transfusion resulted in multi-organ failure and led to mortality in the ICU. These unpleasant experiences played a major role to change our cannulation technique from CC to PC. One patient in the PC group presented large right atrial tear during sternotomy, and instead of repairing it, the mitral valve procedure was completed with transeptal approach through the tear. Another patient with right ventricular injury was easily repaired. In our study, there was no procedure-related mortality in the PC group. The low intracardiac pressure and the absence of cannula in the surgical environment allowed for more convenient repair with peripheral CPB (Figure 2A,B).

Another different finding of this study was that no difference was found between the groups regarding postoperative bleeding, but more RBC was transfused in the CC group (*P*=0.004). After sternotomy, the blood from unexpected injury was not able to be saved in the CC group, a large amount of wastage blood required blood transfusion during the postoperative period. In the PC group, since the CPB was initiated before sternotomy, the blood collected by cardiotomy sucker in the beginning of the operation could be transfused to the patient, thus blood loss was largely avoided.

One study showed that reentry injury and perioperative mortality have a link^[10]^. Our study did not show significant differences in terms of perioperative mortality rates between the groups, although there was a higher occurrence of injury in the CC group. Ellman PI et al.^[11]^ and Imran et al.^[12]^ revealed in their study that reentry injury does not influence long-term survival in redo surgery patients. Patients discharged uneventfully have good long-term survival rates^[11,12]^. In this study, one-year survival rates were found to be excellent and similar between the two groups.

### Limitations of the Study

This is a retrospective non-randomized study and it included limited number of patients. The operation types were not homogeneous between the two groups, which may affect study results. The other important limitations of this study is that the difference in time periods for reoperation may have correlation with cardiac injury, independently of cannulation types, which was not discussed here. Finally, our study results are not reliable enough to give a definite indication for PC in cardiac reoperation. We think that no randomized study can be performed on this topic, and it seems reasonable to evaluate every patient individually before selecting cannulation type.

## CONCLUSİON

Regardless of operation type, presternotomy CPB via PC is associated with lower cardiac injury and less blood transfusion. Peripheral CPB before sternotomy, when feasible, may be especially safer when adhesions of underlying cardiac structures are unknown.

**Table:** 

Authors' roles & responsibilities	
ECA	Substantial contributions to the conception or design of the work; or the acquisition, analysis, or interpretation of data for the work; drafting the work or revising it critically for important intellectual content; final approval of the version to be published
KE	Agreement to be accountable for all aspects of the work in ensuring that questions related to the accuracy or integrity of any part of the work are appropriately investigated and resolved; final approval of the version to be published
MOU	Drafting the work or revising it critically for important intellectual content; final approval of the version to be published
YY	Drafting the work or revising it critically for important intellectual content; final approval of the version to be published
HT	Agreement to be accountable for all aspects of the work in ensuring that questions related to the accuracy or integrity of any part of the work are appropriately investigated and resolved; final approval of the version to be published
SP	Substantial contributions to the conception or design of the work; or the acquisition, analysis, or interpretation of data for the work; final approval of the version to be published
